# Structure basis for the unique specificity of medaka enteropeptidase light chain

**DOI:** 10.1007/s13238-013-0008-x

**Published:** 2014-01-31

**Authors:** Jin Xu, Shi Hu, Xiaoze Wang, Ziye Zhao, Xinyue Zhang, Hao Wang, Dapeng Zhang, Yajun Guo

**Affiliations:** 1 School of Pharmacy, Shanghai Jiao Tong University, Shanghai, 200240 China; 2 International Joint Cancer Institute, Second Military Medical University, Shanghai, 200433 China; 3 State Key Laboratory of Antibody Medicine and Targeting Therapy and Shanghai Key Laboratory of Cell Engineering and Antibody, Shanghai, 201203 China; 4 PLA General Hospital Cancer Center, PLA Postgraduate School of Medicine, Beijing, 100853 China; 5 College of Pharmacy, Liaocheng University, Liaocheng, 252000 China; 6 Medical Biotechnology Institute, Soochow University, Suzhou, 215007 China

**Dear Editor,**

Enteropeptidase (enterokinase, EC 3.4.21.9) is a serine protease, which shows a specific cleavage of its substrates at the C-terminal of the recognition site (Asp)_4_Lys (Zheng et al., 2009). Because of the unique specificity, enteropeptidase could be used as a tool for the production of recombinant fusion proteins. Especially the recombinant enteropeptidase light chain (EPL), which contains a catalytic domain, is of large interest to be applied in biopharmaceutical industry (Lu et al., 1997).

Enteropeptidase has been cloned from several sources, including bovine (Kitamoto et al., 1994), porcine (Matsushima et al., 1994), humans (Kitamoto et al., 1995), mouse (Yuan et al., 1998), and rat (Yahagi et al., 1996). For the high availability, the recombinant bovine enteropeptidase light chain (BEPL) is now most commonly used. However, it is known that BEPL does not exhibit high stringency in its specificity for the canonical target sequence D_4_K (Liew et al., 2007). For instance, Shahravan et al. showed that BEPL also cleaved at unexpected DR and SR sites in AhR6-C/EBP protein (Shahravan et al., 2008). Recently, the enteropeptidase from medaka had been reported to be a more effective tool, because of its much stricter specific for the D_4_K sequence compared to its mammalian counterparts (Ogiwara and Takahashi, 2007). Therefore, we sought to solve the crystal structure of the light chain of medaka enteropeptidase (MEPL), and gain insights into the determinants for its stricter specificity.

MEPL shares high sequence similarity with other EPL classes, which have been structurally identified in previous reports (Fig. S1). Therefore, the crystal structure of MEPL was determined by molecular replacement using the bovine enteropeptidase light chain (PDB entry 1EKB, 53.7% amino acid identity) as the search model and refined to 2.0 Å resolution. The structure of MEPL displays a typical α/β trypsin—like serine protease fold (Fig. 1A). It consists of two six-stranded β-barrels (β1–β6 and β7–β12), either of which makes up about one half of the entire molecule. Both β-barrels are arranged in a Greek-key-pattern containing α-helices at the middle of each barrel with a third α-helix located at the C-terminus. The surface potentials of MEPL reveal an equal distribution of charged amino acids on protein surface although the region near the active center has a predominantly negative potential (Fig. 1B).

**Figure 1:**
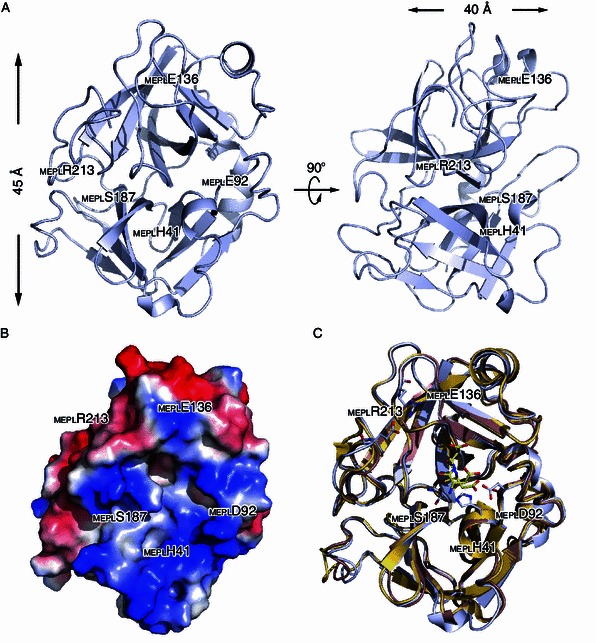
Structure of MEPL. (A) Overall structure of MEPL. The structure of MEPL is shown as ribbon diagrams in two orientations: Front view, looking into the catalytic center; side view, besides the catalytic center. The MEPL molecules are colored as blue-white and several key residues have been labeled. (B) Surface representation and vacuum electrostatic potential of MEPL (where red = electronegative, white = neutral, blue = electropositive). (C) Structural variations of enteropeptidases. A single enteropeptidase molecular of medaka, bovine, and human are shown. The medaka enteropeptidase molecule is colored blue-white, the bovine enteropeptidase is colored salmon, and the human enteropeptidase is colored yellow, respectively. Structural variations are shown with Red Box. Residues in the catalytic center are show in stick. The residues have been renumbered according to the sequence of MEPL

Superimposing of MEPL with the bovine enteropeptidase light chain resulted in an rmsd of 0.79 Å for the C^a^ coordinates (Fig. 1C). The differences are mainly located in the loop regions, such as the so-called ‘131-loop’ that connects strand β7 and β8. MEPL also contains, relative to bovine enzyme, an additional small 3_10_-helix between the strand β4 and β5. Furthermore, the catalytic triad of the unliganded medaka enteropeptidase superimposes well with the bovine enzyme in complex with a trypsinogen-activation peptide analogue (Lu et al., 1999). However, in secondary structure, the active centers do not show any obvious differences between the two enzymes, which intimates that the stricter specificity of MEPL may depend on the distinct amino acid sequence and unique intramolecular interactions.

The comparison of the catalytic centers between MEPL, BEPL and HEPL (human enteropeptidase light chain, PDB entry 4DGJ, amino acid identity 55%), provide an understanding of the mechanism of substrate specificity functioned by these different enzymes (Fig. 2A and 2B).

**Figure 2:**
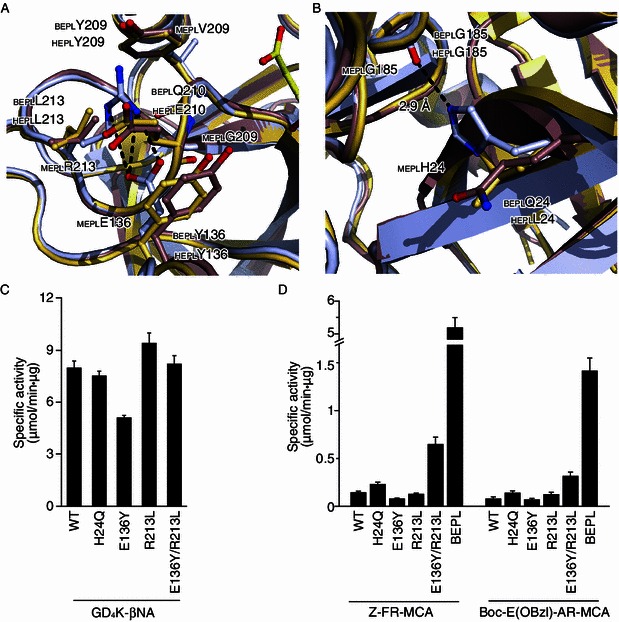
Comparison of the catalytic center in MEPL, BEPL and HEPL, and specific assay of MEPL variants. (A and B) View of detailed two regions considered as playing crucial role in the specificity mechanism. Key residues are shown as colored sticks (blue-white MEPL, salmon BEPL and yellow HEPL, respectively). (C) Recombinant MEPL variants were assayed by using GD4K-βNA as specific substrate. (D) Recombinant MEPL variants were assayed by using Z-FR-MCA and Boc-E(OBzl)-AR-MCA as unspecific substrates

In the structure of BEPL with a trypsinogen-activation peptide (VD_4_K), the side chain of Lys-P1 inserts deeply into a specific pocket, at the bottom of which Asp181 neutralizes the terminal amino group (amino acid residues of peptidyl substrates customarily are numbered P1, P2, P3, etc. (Schechter and Berger, 1967)). The structure of this cleft determinates that the P1 site of substrates could only be lysine or arginine (Lu et al., 1999). This specific pocket consists of three parts: the strand β11, the so called “174-loop” that connects strand β9 and β10, and the “208-loop” that connects strand β11 and β12. The main differences locate at the N-terminus of “208-loop”, while the sequences of strand β11 and “174-loop” are highly conservative. Compared with BEPL and HEPL, MEPL shows much less activity for the peptide substrates with arginine at P1 site, which indicates its restriction for the entrance of arginine residues. And according to the structural details, we supposed that the variations of _MEPL_H24, _MEPL_E136, _MEPL_V209, _MEPL_G210 and _MEPL_R213 may play curial role in the tension changes of the loop chains around the pocket of catalytic center.

In MEPL structure, _MEPL_E136 makes three hydrogen bonds with _MEPL_R213, while there are no similar interactions in BEPL because it is _BEPL_L213, _BEPL_Y136 in these positions as well as the same situation for the HEPL structure (Fig. 2A). Although the interactions are a little far distance from the catalytic center (8 Å), these hydrogen bonds may help anchoring the “208-loop”, restricting the electron density size of substrate. In addition, _MEPL_V209 and _MEPL_G210 with short side chains are covered and fixed by the side chains of four residues (_MEPL_E136, _MEPL_E163, _MEPL_R213 and _MEPL_R216), reducing the mobility of “208-loop” and strand β11 in MEPL. However, the amino acid residues at the same positions in BEPL and HEPL are exposed on the surface of the proteins, and therefore possess higher flexibility (Figs. 2A and S2). Moreover, we observed that the imidazole group of _MEPL_H24 could form a hydrogen bond with the main chain oxygen atom of _MEPL_G185, which could help improving the structural rigidity of “174-loop” (Fig. 2B). Nevertheless, there are no such interactions in BEPL and HEPL because it is _BEPL_Q24 and _HEPL_L24 at this position, respectively. Over all, the unique residue interactions in MEPL may reduce the flexibility of the specific pocket, restricting the entrance of arginine residue which has larger side chain.

In order to study the impacts of these residues on MEKL-substrate interaction, 3 residues in MEPL were replaced by those in BEPL respectively (H24Q, E136Y and R213L), and a combined mutant E136Y/R213L was built. Then, the specific activities of the four variants were assayed by using the specific substrate GD_4_K-βNA and the non-specific substrates Boc-E(OBzl)-AR-MCA and Z-FR-MCA. The results showed that three mutants (H24Q, R213L and E136Y/R213L) exhibited significantly increased activity for the MCA-containing substrates, while retaining GD_4_K-βNA hydrolyzing activity (Fig. 2C and 2D). This result indicated the importance of the two interactions (H24-G185 and E136-R213) for the specificity of enteropeptidase. Nevertheless, these mutations did not raise the activities of MEPL on unspecific substrates to the level of BEPL, which meant there should be other facts affecting the substrate-selectivity of MEPL. The low enzyme activity of mutant E136Y for both GD4K-βNA and the MCA-containing substrates meant that the amino acids in position 136 might affect the activity of enteropeptidase. And it was also been provided by the kinetic studies (Table S3).

Taken together, our data on cleavage of peptide substrates, faithfully supported by crystal structures, present an extraordinary example of fine adjustment of enzyme mechanism. Of note, this finding can be directly applied in the reengineering of other enteropeptidases, for instance BEPL and HEPL, to improve the specificity.

## Electronic supplementary material

Below is the link to the electronic supplementary material.

Supplementary material 1 (PDF 553 kb)
