# Supplementary material for: Structure basis for the unique specificity of medaka enteropeptidase light chain
Source: Protein Cell. 2014 Jan 31;5(3):178–81. doi: 10.1007/s13238-013-0008-x (PMC3967055; doi:10.1007/s13238-013-0008-x)
Supplement: Supplementary file 1 — Supplementary material 1 (PDF 553 kb) [file 13238_2013_8_MOESM1_ESM.pdf]

Supplementary data

Figure. S1

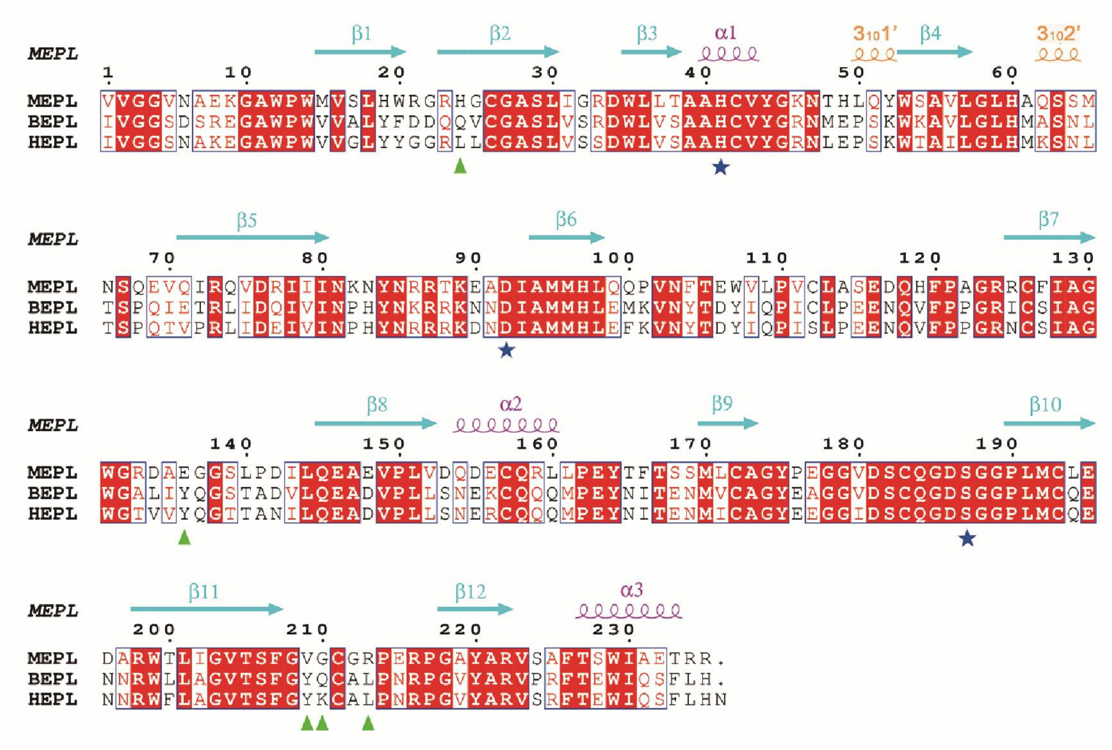

Figure S1. Amino acid sequence alignments of the EPL from different sources. Amino acid residues are numbered based on the sequence of MEPL (top number). Secondary structure elements of MEPL are labeled above the sequence. The active-site residues (H41, D92 and S187) are indicated by blue star. The residues, which are expected to be the determinants for the stricter specificity of MEPL, are indicated by green triangles.

Figure. S2

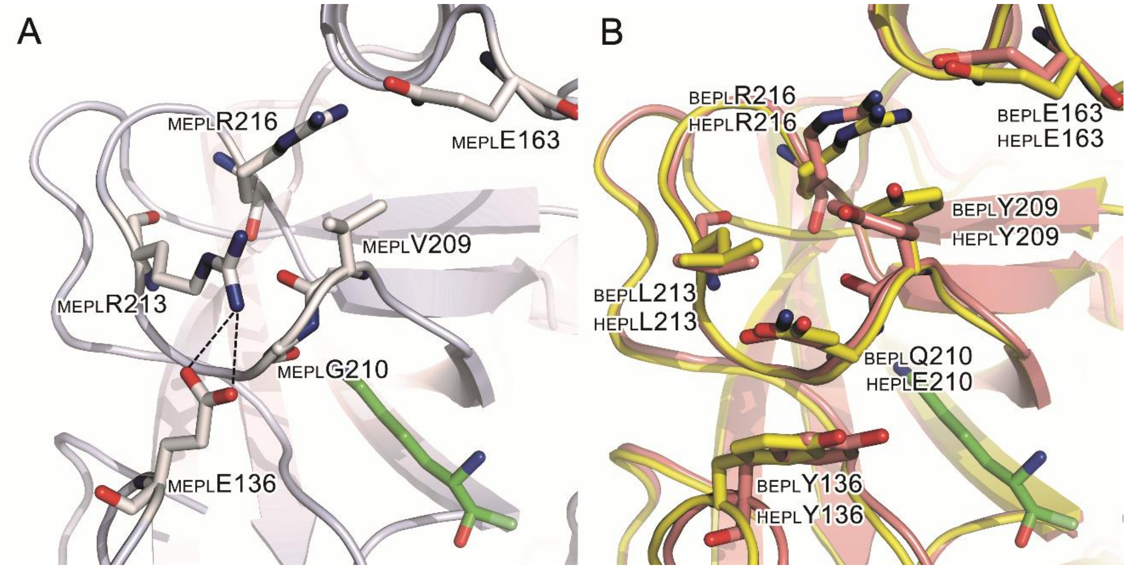

Figure S2. Comparison of the substrate binding exosites between MEPL (ice blue), BEPL (pink) and HEPL (yellow). MEPL and HEPL were modeled in complex with an analog of the trypsinogen activation peptide reported by *Lu D etc*. The substrate Asp-Asp-Asp-Lys was shown in stick-model in green; the key residues are shown in stick-model (ice blue MEPL, pink BEPL and yellow HEPL, respectively).

Table S1 Structural Parameters

**Data collection**	
Space group	$P2_12_12_1$
Cell parameters	
a, b, c (Å)	48.5, 71.65, 134.5
α, β, γ (°)	90, 90, 90
Wavelength used (Å)	1.0000
Resolution (Å)	50.0 (2.7) – 2.0
No. of all reflections	225834(11047.6)
No. of unique reflections	32262(1556)
Completeness (%)	99.7 (100)
Average I/σ(I)	28.5 (8.2)
R_{merge}^{a} (%)	7.7 (32.3)
**Refinement**	
No. of reflections used (σ(F) > 0)	32203
R_{work}^{b} (%)	18.9
R_{free}^{b} (%)	24.0
r.m.s.d. bond distance (Å)	0.008
r.m.s.d. bond angle (°)	1.249
Average overall *B*-value (Å^2)	25.3
Ramachandran plot	
Res. in most favored regions	672 (88.1%)
Res. in additionally allowed regions	91(11.9 %)
PDB ID	3W94

$^{a}R_{merge} = \Sigma_h\Sigma_l \mid I_{ih} - \langle I_h \rangle \mid / \Sigma_h\Sigma_I \langle I_h \rangle$, where $\langle I_h \rangle$ is the mean of multiple observations I_{ih} of a given reflection h.

$^{b}R_{work} = \Sigma||F_p(obs)|-|F_p(calc)||/ \Sigma|F_p(obs)|$; R_{free} is an R-factor for a selected subset (5%) of reflections that was not included in prior refinement calculations.

^{C}Numbers in brackets: the values for the outer resolution shell.

Table S2. PCR primers used for mutant MEPL production by site-directed mutagenesis.

Mutations	Primers
H24Q	5'- AATCAGGCTCGCGCCGCAGCCTTG -3'
	5'- CAAGGCTGCGGCGCGAGCCTGATT -3'
E136Y	5'- CAGGCTGCCGCCATACGCAT -3'
	5'- GATGCGTATGGCGGCAGCCT -3'
R213L	5'- GGAAGGCCGCAGCCCACGCCAAAG -3'
	5'- CGTGGGCTGCGGCCTTCCG -3'

Table S3. Kinetic parameters for the cleavage of substrates GD_4K-βNA, Boc-E(OBzl)-AR-MCA and Z-FR-MCA

Protease	**GD_4K-βNA**[a]			**Boc-E(OBzl)-AR-MCA**[b]			**Z-FR-MCA**[c]		
	Km, mM	*kcat*, Min^{-1}	*kcat/Km* mM× min^{-1}	*Km*, mM	*kcat*, Min^{-1}	*kcat/Km* mM× min^{-1}	*Km*, mM	*kcat*, Min^{-1}	*kcat/Km* mM× min^{-1}
WT	0.6	1672	2618	1.5	20.5	13.5	1.6	38.5	24.6
H24Q	0.4	1572	3787	0.7	35.9	50.7	1.1	60.4	53.1
E136Y	0.6	1070	1778	1.3	18	13.9	1.6	21.3	13
R213L	0.5	1981	3663	0.8	32	40.2	0.9	33.3	37
E136Y/R213L	0.3	1725	5968	0.6	83	137.2	1.6	170.6	108.7
BEPL	0.7	1558	2226	0.2	204	1020	0.5	1539	3078

a GD4K, Gly-Asp-Asp-Asp-Asp-Lys; βNA, β-naphthylamine.

b Boc, t-butyloxycarbonyl; OBzl, benzyloxycarbonyl(butanoil); AR, Ala-Arg.

c Z, benzyloxycarbonyl; FR, Phe-Arg; PFR.

Material and Methods

Construction of recombinant Escherichia coli for expression of MEPL

The MEPL-encoding DNA fragment was synthesized and cloned into the expression vector pET-32 (Novagen) at a site downstream to the fusion partner thioredoxin (Trx) gene, following the sequence encoding D_4K. The expression plasmid pET-32-MEPL was transformed into *E.coli* BL21(DE3).

Protein expression and purification

The medaka enteropeptidase light-chain sequence was taken from the Uni-Prot database (ID A4UWM5; residues range from 795 to 1036). The nucleotide sequence was synthesized and cloned into the expression vector pET-32 (Novagen) at the endonuclease *Kpn* I and *Bam* HI sites. The final construct encoded an N terminal Trx-tag, a 5-amino acid linker containing an enteropeptidase cleavage site, and MEPL.

The protein was overexpressed in an *E.coli* strain BL21 (DE3) as inclusion bodies. The expression was induced by the addition of 0.5 m*M* IPTG when OD_{600} reached 0.6. Cells were harvested by centrifugation and the pellets were suspended in 20 m*M* Tris (pH = 8.0) and lysed by sonication. The cell lysate was then centrifuged to get the precipitate. Then, the inclusion bodies were solubilized by the solution containing 8.5 *M* Urea and 20 m*M* β-mercaptoethanol for 3 h and ultra-centrifuged at 10,000 g for 1h. The refolding was performed by a fast dilution method in 20 m*M* $NH_3 \cdot H_2O$ and 10% Glycerol at pH = 10.5. After 72 h at 4 ℃, the refolding solution was dialyzed to 20 m*M* Tris (pH = 8.0) at

room temperature to facilitate autocatalytic activation by cleaving the fusion Trx-tag at the D_4K sequence.

MEPL was further purified by affinity chromatography on STI-agarose (Sigma). The column was equilibrated with 20 m*M* Tris (pH 8.0) and 50 m*M* NaCl and then later eluted with 50 m*M* HCOONa (pH 3.0). The final sample of MEPL was dialyzed against 10 m*M* Tris-HCl, 150 m*M* NaCl, pH = 8.0, and concentrated to 20 mg mL^{-1}. The determination of protein concentrations was carried out spectrophotometrically by absorbance at 280nm using a molar extinction coefficient of 59,970 M^{-1} cm^{-1} calculated from the known amino acid sequence.

Crystallization

The concentrated protein sample (20 mg mL^{-1} in 10 m*M* Tris-HCl, 150 m*M* NaCl, pH = 8.0) was screened for crystallization using commercial available screen kits. Following extensive optimization trials, the crystallization was performed at 291 K using the hanging-drop vapor-diffusion technique. The hanging drops were obtained by mixing 1-µl of the protein solution with 1-µl reservoir buffer containing 20% polyethylene glycol (PEG) 3,350, 0.1 *M* cadmium chloride, 0.1 *M* sodium acetate (pH 4.6), and equilibrated against 500-µl reservoir solution. The protein crystals reached final dimensions of 100×100×100 μm^3 with the best diffraction within one week.

For data collection, the native crystals were soaked in cryoprotectant (20% PEG 3,350, 0.1 *M* cadmium chloride, 0.1 *M* sodium acetate pH 4.6, and 5% glycerol), and flash-cooled in liquid nitrogen. Then the crystals were transferred into a dry nitrogen stream at 100 K for X-ray data collection.

Data collection, structure determination, and refinement

X-ray diffraction data were collected at beamline BL17A (Photon Factory, Japan) at a resolution of 2.0 Å. Data were processed, integrated, and scaled using the HKL2000 program package (Otwinowski and Minor, 1997). The crystals belonged to space group $P2_12_12_1$ with unit-cell dimensions a = 48.5 Å, b = 71.65 Å, and c = 134.5 Å.

The structure of MEPL was solved by molecular replacement method, employing the crystal structures of BEPL (PDB entry 1EKB) (Lu et al., 1999) as the initial searching model by using the program PHASER (McCoy et al., 2005). The clear solutions in both the rotation and translation functions indicated the presence of two molecules in one asymmetric unit, which is consistent with the Matthews coefficient and solvent content (Matthews, 1968). The disconsistent residues were manually rebuilt in the program Coot under the guidance of the *Fo-Fc* and 2*Fo-Fc* electron density maps (Emsley and Cowtan, 2004). After the refinement in PHENIX (Adams et al., 2002), the respective working Rfactor and Rfree dropped from 0.42 and 0.48 to 0.19 and 0.24, respectively, for all data from 50.0 to 2.0 Å. Refinement was monitored by calculating Rfree based on a subset containing 5% of the total reflections. Model geometry was verified using the program PROCHECK (Laskowski et al., 1993). Data collection and refinement statistics are detailed in Table S1. All structure figures were prepared using PYMOL (DeLano, 2002).

Site-directed mutagenesis

Site-directed mutagenesis was carried using overlap-PCR to produce the corresponding fragments for the following mutants: H24L, Y136E, R213L, and Y136E/R213L. The DNA of wide type (WT) MEPL was used as the template and the

primers used to construct MEKL variants were shown in Table S2. The PCR products were digested with KpnI and BamHI (Takara Biotech), gel purified, and ligated into the expression vector pET-32. All mutations were confirmed by DNA sequencing and correct plasmids for mutants were transformed into E.coli BL21 (DE3).

Enzyme assays

The activities of the recombinant MEPL variants and BEPL were determined by using the specific substrate GD4K-βNA (Sigma, St. Louis, MO) according to the method described before (Ogiwara and Takahashi, 2007). Enzyme activity for the non-specific substrates Boc-E(OBzl)-AR-MCA and Z-FR-MCA (Peptide Institute, Osaka, Japan) were measured by the method of Barrett (Barrett, 1980). These assays were repeated three times.

Kinetic Studies

The kinetic assay of EP for GD_4K-βNA was performed at 30℃ in 100ul buffer containing 0.04 – 0.8 mM GD_4K-βNA, 25 mM Tris (pH 8.3), 2.5% dimethylsulfoxide and 2 mM $CaCl_2$. The reaction was initiated by adding MEPL (final concentration 18.7 nM). The active rate was determined from the continuously increment of fluorescence (λ_{ex} = 337 nm and λ_{em} = 420 nm) for 3min. The *Km* and *kcat* were determined from Lineweaver-Burk plots. These assays were repeated three times.

The kinetic assay for various 4-methylcoumaryl-7-amide (MCA)-containing peptide substrates was determined in 100 ul buffer containing 25 mM PB (pH7.5), 2.5% dimethylsulfoxide and 2 mM Ethylenediaminetetraacetic acid disodium salt (EDTA),

with concentration range of the substrates 0.08 – 0.8mM. Reactions were started by addition of MEPL (final concentration 37 nM) and fluorescence absorbance (λ_{ex} = 380 nm and λ_{em} = 460 nm) of the released MCA was monitored continuously for 3 min. The *Km* and *kcat* were determined from Lineweaver-Burk plots. These assays were repeated three times.

References

Adams, P.D., Grosse-Kunstleve, R.W., Hung, L.W., Ioerger, T.R., McCoy, A.J., Moriarty, N.W., Read, R.J., Sacchettini, J.C., Sauter, N.K., and Terwilliger, T.C. (2002). PHENIX: building new software for automated crystallographic structure determination. Acta Crystallogr D Biol Crystallogr 58, 1948-1954.

Barrett, A.J. (1980). Fluorimetric assays for cathepsin B and cathepsin H with methylcoumarylamide substrates. Biochem J 187, 909-912.

DeLano, W. (2002). The PyMOL Molecular Graphics System

Emsley, P., and Cowtan, K. (2004). Coot: model-building tools for molecular graphics. Acta Crystallogr D Biol Crystallogr 60, 2126-2132.

Laskowski, R., MacArthur, M., Moss, D., and Thornton, J. (1993). PROCHECK: a program to check the stereochemical quality of protein structures. J Appl Cryst 26, 283-291.

Lu, D., Futterer, K., Korolev, S., Zheng, X., Tan, K., Waksman, G., and Sadler, J.E. (1999). Crystal structure of enteropeptidase light chain complexed with an analog of the trypsinogen activation peptide. J Mol Biol 292, 361-373.

Matthews, B.W. (1968). Solvent content of protein crystals. J Mol Biol 33, 491-497.

McCoy, A., Grosse-Kunstleve, R., Storoni, L., and Read, R. (2005). Likelihood-enhanced fast translation functions. Acta Crystallogr D Biol Crystallogr 61(Pt 4),458-464.

Ogiwara, K., and Takahashi, T. (2007). Specificity of the medaka enteropeptidase serine protease and its usefulness as a biotechnological tool for fusion-protein cleavage. Proc Natl Acad Sci U S A 104, 7021-7026.

Otwinowski, Z., and Minor, W. (1997). Processing of X-ray diffraction data collected in oscillation mode. In Macromolecular Crystallography, part A, C.W. Carter Jr., and R.M. Sweet, eds. (Academic Press), pp. 307-326.
